# Association of treatment method with mortality and functional outcome in patients with displaced femoral neck fracture: a cohort study of 60–69-year-old patients from the Swedish Hip Fracture Register

**DOI:** 10.2340/17453674.2026.46368

**Published:** 2026-09-03

**Authors:** Pierre CAMPENFELDT, Margareta HEDSTRÖM, Olle OLOFSSON, Karin MODIG, Amer AL-ANI

**Affiliations:** 1 Department of Clinical Science, Intervention and Technology (CLINTEC), Karolinska Institutet, Stockholm; 2 Trauma and Reparative Medicine Theme (TRM), Karolinska University Hospital, Stockholm; 3 Sundsvall Hospital, Sundsvall; 4 Institute of Environmental Medicine, Unit of Epidemiology, Karolinska Institutet, Stockholm; 5 ArtroFotkliniken AB, Stockholm, Sweden

## Abstract

**Background and purpose:**

Optimal treatment for displaced femoral neck fractures (FNF) in patients aged 60–69 years remains controversial. We aimed to compare mortality and short-term clinical outcomes after total hip arthroplasty (THA), hemiarthroplasty (HA), or internal fixation (IF) after displaced FNF.

**Methods:**

We included 3,325 patients aged 60–69 years with displaced FNF recorded in the Swedish Hip Fracture Register. 1-year mortality and 4-month functional outcomes (maintenance of independent living and outdoor walking ability) were analyzed. Analyses were stratified by American Society of Anesthesiologists (ASA) class (I–II vs III–IV).

**Results:**

Overall, 1-year mortality was 8.3%. Adjusted analyses showed higher 1-year mortality after HA (odds ratio [OR] 2.5, 95% confidence interval [CI] 1.7–3.6) and IF (OR 2.3, CI 1.6–3.3) compared with THA. Survival analyses demonstrated lower survival probabilities after HA and IF compared with THA in ASA III–IV but not in ASA I–II patients. THA was associated with higher odds of maintaining independent living (IF: OR 0.40, CI 0.20–0.86; HA: OR 0.09, CI 0.04–0.17) and maintaining outdoor walking ability (IF: OR 0.35, CI 0.24–0.50; HA: OR 0.26, CI 0.16–0.43) in ASA I–II patients. In ASA III–IV patients, estimates were closer to unity.

**Conclusion:**

THA was associated with lower mortality in ASA III–IV and better functional outcomes in ASA I–II.

There is broad agreement that patients ≥ 70 years with displaced femoral neck fracture (FNF) are best managed with arthroplasty due to the high rate of complications after internal fixation (IF) [1–3]. The choice between hemiarthroplasty (HA) and total hip arthroplasty (THA) in older patients remains debated; HA is commonly selected for those with limited life expectancy, cognitive impairment, or low activity levels [4-6]. In younger patients, IF has traditionally been preferred to preserve the femoral head and support higher activity. However, in patients aged 60–69 years with displaced femoral neck fractures, THA has become an established treatment option and is widely used in current clinical practice [7–9]. A systematic review of 16 randomized trials including 3,084 patients aged > 50 years reported similar outcomes between HA and THA with respect to revision, function, mortality, and dislocation up to 5 years [10].

Evidence specific to patients aged 60–69 years remains limited, particularly regarding short-term mortality and functional recovery after IF, HA, and THA. Therefore, we aimed to compare mortality and clinical outcomes among patients aged 60–69 years with displaced FNF treated with IF, HA, or THA.

## Methods

### Study design

This nationwide, registry-based cohort included patients aged 60–69 years with non-pathological displaced femoral neck fractures (Garden III–IV) [11] admitted between January 1, 2013 and December 31, 2019 and recorded in the Swedish Hip Fracture Register (SHR; Rikshöft) [8]. During the study period, the SHR covered approximately 80–86% of all hip fractures in Sweden [12]. Previous studies have demonstrated good agreement between the SHR and the Swedish National Patient Register regarding hip fracture registration, supporting the validity and representativeness of the registry for epidemiological research [13]. Baseline data and 4-month follow-up were retrieved from SHR. Baseline data completeness was high for most variables used in the analyses, with less than 3% missing data for residency, walking ability, and ASA classification. Cognitive status had a higher proportion of missing values (approximately 28%) and was therefore interpreted with caution. Mortality data was obtained through linkage with the Swedish Cause of Death Register, providing complete follow-up for mortality analyses.

**Figure 1:**
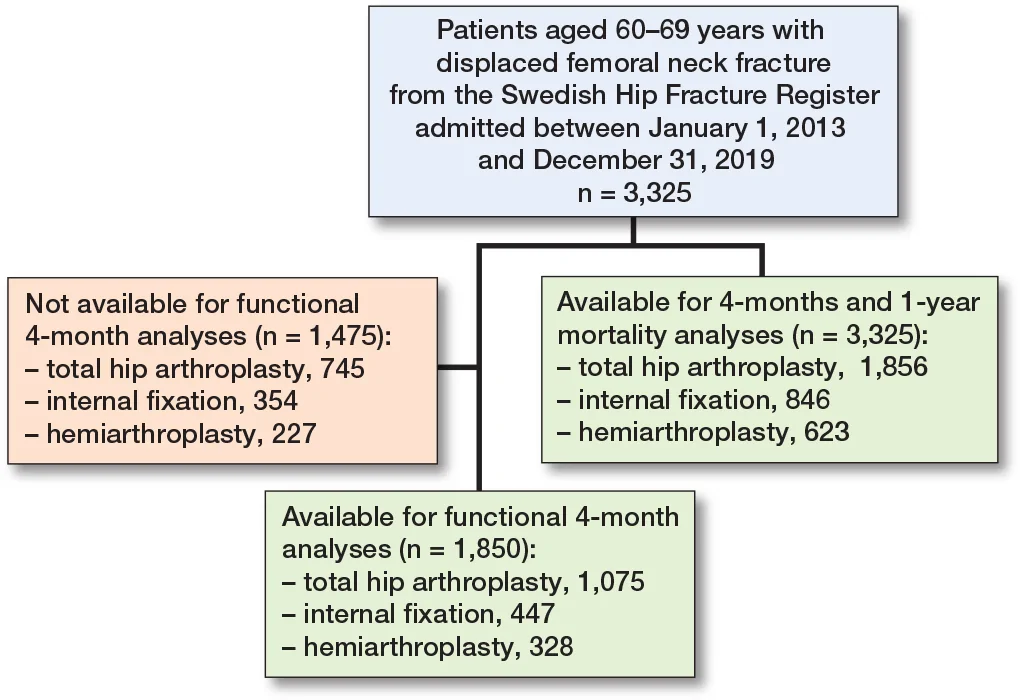
Flow diagram of patient inclusion and follow-up. Of the 1,475 patients not available for functional 4-months analysis, 149 died before follow-up and 1,326 were non-responders. Compared with responders, non-responders generally had poorer baseline functional status, higher ASA class, and more cognitive impairment

Functional outcomes were assessed 4 months after surgery using the routine SHR follow-up questionnaire [14]. Follow-up data was available for 1,850 of 3,325 patients (56%). Of the remaining patients, 149 had died before the 4-month follow-up and the remainder were non-responders (see Figure 1). The study is reported in accordance with STROBE guidelines [15].

### Data categorization

Baseline variables included age, surgical method, sex, time to surgery (hours), length of stay (days), residency (independent or dependent living), walking ability (outdoor, indoor, or unable), walking aids (no/1 aid, 2 aids, walker or wheelchair/in bed), comorbidity (ASA class), and cognitive status (fully oriented, partially oriented, dementia). Continuous variables are reported as mean (standard deviation [SD]), and categorical variables as number (percentage).

Follow-up variables at 4 months included residency, walking ability, and walking aids. Mortality was assessed at 4 and 12 months. Surgical procedure was categorized as IF (1/2/3 screws/pins/nails), HA, or THA. Other methods were excluded. Residency was categorized as independent living (own home or service home) or dependent living (institutional care, rehabilitation unit, or other). Walking ability was categorized as: (i) outdoor walking, (ii) indoor only, or (iii) unable to walk. Walking aids were categorized as: (i) none/1 aid, (ii) 2 aids/walker, or (iii) wheelchair/cannot walk. Comorbidity was defined using the American Society of Anesthesiologists (ASA) classification [16], which is widely used in hip fracture research to predict prognosis [10]. ASA ranges from I to V, with lower scores indicating better health. For analysis, patients were grouped as ASA I–II and ASA III–IV, as no patients were classified as ASA V.

### Outcomes

Outcomes included mortality and functional outcomes. Mortality was assessed at 4 and 12 months. Functional outcomes at 4 months comprised maintenance of independent living, maintenance of outdoor walking ability, and wheelchair dependence. Maintenance of independent living was defined as living independently at follow-up among patients who were living independently before fracture. Maintenance of outdoor walking ability was defined as the ability to walk outdoors at follow-up among patients who had this ability before fracture. Wheelchair dependence was defined as reliance on a wheelchair at follow-up. These outcomes reflect different dimensions of functional recovery.

### Statistics

Because treatment allocation was not randomized and was strongly influenced by baseline health, analyses were performed using an estimation-based framework.

Baseline characteristics are presented descriptively by surgical method (THA, HA, IF). No statistical testing was performed for baseline comparisons. Outcomes were analyzed using logistic regression to estimate odds ratios (ORs) with 95% confidence intervals (CIs). THA was used as the reference category due to its clinical role as the most functionally demanding procedure.

Formal interaction testing between ASA class and surgical method was performed for all functional outcomes and mortality. Given the strong association between ASA class, treatment selection, and outcome, analyses were stratified by ASA class (I–II and III–IV). This stratification was prespecified and intended to improve clinical interpretability rather than to identify causal subgroup effects.

Mortality was analyzed using 2 complementary approaches. Adjusted logistic regression estimated cumulative mortality at predefined time points (4 and 12 months), whereas Kaplan–Meier analysis evaluated time-to-event data by accounting for the timing of events and censoring throughout follow-up. Because these methods estimate different aspects of mortality, they may yield different results. Logistic regression estimates the probability of death at fixed time points, whereas Kaplan–Meier analysis requires a sustained divergence of survival curves over time to demonstrate differences in survival. Analyses were performed in SPSS version 28 (IBM Corp, Armonk, NY, USA).

### Ethics, data sharing, funding, use of AI, and disclosures

The study conformed to the Declaration of Helsinki [17] and was approved by the Swedish Ethical Review Authority (Dnr 2017-71088-31 and Dnr 2020-04075).

The data underlying this study was obtained from the Swedish Hip Fracture Register and the Swedish National Board of Health and Welfare under ethical approval and data-sharing agreements. The data is not publicly available due to Swedish data protection legislation but may be available from the respective data holders upon reasonable request and with appropriate ethical and legal approvals. No external funding was received for this study.

ChatGPT (OpenAI, San Francisco, CA, USA) was used to assist with language editing and improvement of English grammar and readability. The authors reviewed and approved all text for the final manuscript.

The authors declare no competing interests. Complete disclosure of interest forms according to ICMJE are available on the article page, doi: 10.2340/17453674.2026.46368

## Results

3,325 patients aged 60–69 years with displaced FNF were included (Figure 1). THA was performed in 56% of patients, IF in 25%, and HA in 19%. Baseline characteristics showed that patients treated with HA had higher comorbidity, severe cognitive status, and poorer baseline function (Table 1).

**Table 1:** Baseline characteristics per surgical method of all included patients and for patients available at 4-month follow-up. Values are number (%) unless otherwise specified

Factor	Full sample (n = 3,325)			4-month sample (n = 1,850)		
	THA	IF	HA	THA	IF	HA
	n = 1,856	n = 846	n = 623	n = 1,075	n = 447	n = 328
Female	1,030 (56)	535 (63)	353 (57)	596 (55)	296 (66)	182 (55)
Mean age (SD)	66 (2.7)	66 (2.7)	66 (2.7)	66 (2.7)	66 (2.6)	66 (2.7)
Waiting time (SD) **^a^**	27 (26)	16 (16)	28 (28)			
Hospital stay (SD) **^b^**	6.9 (4.3)	5.7 (4.9)	8.3 (5.7)			
Residency						
Independent living	1,765 (98)	791 (97)	575 (94)	1,072 (99)	438 (98)	313 (96)
Dependent living	44 (2.4)	29 (3.5)	34 (5.6)	2 (0.2)	8 (1.8)	13 (4.0)
Missing	47	26	14	1	1	2
Walking ability						
Outdoor	1,698 (93)	755 (90)	514 (83)	1,055 (98)	428 (96)	292 (89)
Indoor	91 (5.0)	57 (7.0)	61 (10)	14 (1.2)	16 (3.6)	27 (8.0)
Unable to walk	40 (2.0)	29 (3.0)	42 (7.0)	2 (0.2)	2 (0.4)	8 (3.0)
Missing	27	5	6	4	1	1
Walking aid						
No or one aid	1,516 (83)	686 (83)	436 (71)	925 (86)	367 (83)	240 (75)
Two aids or walker	292 (16)	123 (15)	145 (24)	146 (14)	73 (16)77 (24)	
Wheelchair/ in bed	26 (1.0)	21 (3.0)	31 (5.0)	1 (-)	2 (1.0)	3 (1.0)
Missing	22	13	8	3	5	8
Comorbidity						
ASA I–II	1,201 (65)	526 (63)	189 (31)	753 (71)	297 (67)	106 (33)
ASA III–IV	638 (35)	305 (37)	429 (69)	314 (29)	144 (33)	218 (67)
Missing	17	15	5	8	6	4
ASA class						
I	288 (16)	187 (22)	22 (4.0)	191 (18)	125 (28)	9 (3.0)
II	913 (50)	339 (41)	167 (27)	562 (53)	172 (39)	97 (30)
III	601 (33)	254 (31)	365 (59)	299 (28)	118 (27)	193 (59)
IV	37 (2.0)	51 (6.0)	64 (10)	15 (1.0)	26 (6.0)	25 (8.0)
Missing	17	15	5	8	6	4
Cognitive status						
Fully oriented	1,224 (91)	522 (87)	362 (81)	747 (92)	298 (91)	199 (80)
Partially oriented	61 (5.0)	42 (7.0)	40 (9.0)	33 (4.0)	13 (4.0)	22 (9.0)
Dementia	59 (4.0)	34 (6.0)	45 (10)	30 (4.0)	15 (5.0)	27 (11)
Missing	512	248	176	265	121	80

a Mean waiting time (hours).

b Mean length of hospital stays (days).

IF = internal fixation; HA = hemiarthroplasty; THA = total hip arthroplasty; SD = standard deviation; ASA= American Society of Anesthesiologists.

### Mortality

Overall mortality was 4.5% at 4 months and 8.3% at 1 year. Mortality was lowest among patients treated with total hip arthroplasty (THA) and highest among those treated with hemiarthroplasty (HA), with internal fixation (IF) showing intermediate values (Table 2).

**Table 2:** Functional outcome at 4 months and mortality at 4-month and 1-year follow-up for patients treated with THA, IF, or HA. Values are count and (%) or unadjusted and adjusted odds ratio (OR) with 95% confidence interval (CI) using binary logistic regression analysis

	Total n	THA	IF	HA	IF vs THA OR (CI)	HA vs THA OR (CI)
Functional outcome at 4 months	1,850	1,075 (58)	447 (24)	328 (18)		
Maintained independent living	1,708 (92)	1,036 (97)	407 (93)	265 (84)	0.43 (0.27–0.69) **^a^**	0.19 (0.12–0.30) **^b^**
					0.42 (0.26–0.68) **^b^**	0.22 (0.13–0.35) **^b^**
Maintained outdoor walking ability	1,426 (77)	885 (84)	310 (72)	231 (78)	0.52 (0.39–0.68) **^a^**	0.69 (0.50–0.96) **^a^**
					0.54 (0.41–0.70) **^b^**	0.92 (0.66-1.3) **^b^**
Wheelchair dependence	114 (6.2)	37 (4.0)	42 (9.0)	35 (11)	2.9 (1.8–4.5) **^a^**	3.4 (2.1–5.5) **^a^**
					2.9 (1.8–4.6) **^b^**	2.9 (1.7–4.8) **^b^**
Mortality	3,325	1,856 (56)	846 (25)	623 (19)		
At 4 months	149 (4.5)	40 (2.0)	56 (7.0)	53 (9.0)	3.2 (2.1–4.9) **^a^**	4.2 (2.8–6.4) **^a^**
					3.0 (1.9–4.9) **^c,d^**	2.7 (1.6–4.5) **^c,d^**
At 1 year	276 (8.3)	94 (5.0)	94 (11)	88 (14)	2.3 (1.7–3.1) **^a^**	3.1 (2.3–4.2) **^a^**
					2.3 (1.6–3.3) **^c,d^**	2.5 (1.7–3.6) **^c,d^**

For abbreviations, see Table 1.

a Unadjusted.

b Adjusted for age, sex and ASA.

c Adjusted for age, sex, ASA and mental state.

d Missing = 961 (29%).

In adjusted logistic regression analyses (adjusted for age, sex, and cognitive status), HA and IF were associated with higher odds of mortality at both 4 months and 1 year compared with THA (see Table 2). These patterns were also observed in analyses stratified by ASA class.

**Figure 2:**
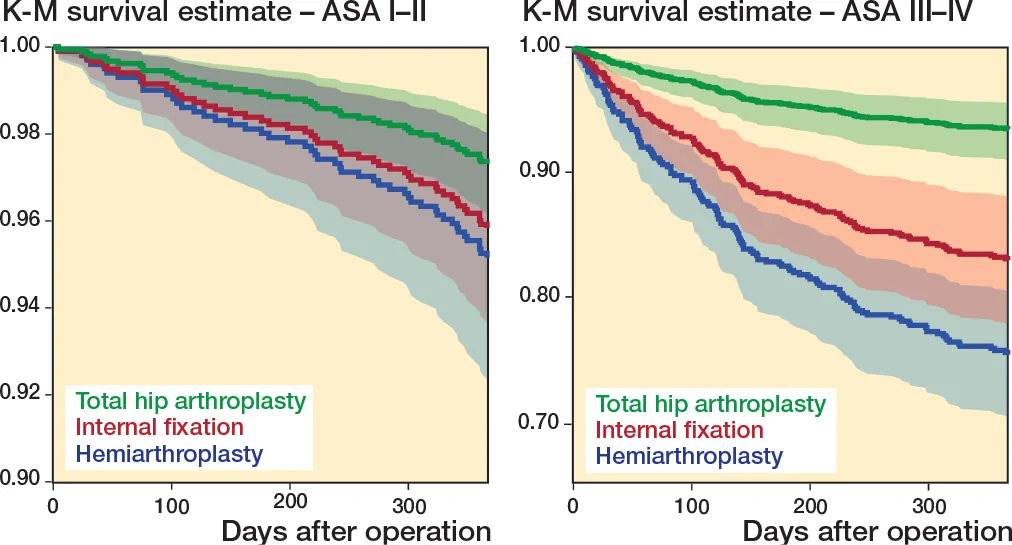
Kaplan–Meier curves for 1-year mortality stratified by ASA class. Among patients with ASA I–II, survival curves were similar across surgical methods. Among patients with ASA III–IV, survival probabilities were lower for patients treated with HA, with IF showing intermediate estimates and THA the highest survival over follow-up. Shaded areas represent 95% confidence intervals.

In time-to-event analyses using Kaplan–Meier methods, adjusted for age, sex, and cognitive status, survival curves were similar across surgical methods among patients with ASA I–II, with overlapping confidence intervals throughout follow-up. In contrast, among patients with ASA III–IV, survival probabilities were consistently lower for patients treated with HA, with IF showing intermediate estimates and THA the highest survival over time (Figure 2).

### Functional outcomes at 4 months

Functional outcomes at 4 months showed that patients treated with THA more frequently maintained independent living and outdoor walking ability, and less often relied on a wheelchair, compared with IF and HA. In adjusted 3-group analyses (see Table 2), both IF and HA were associated with lower odds of favorable functional outcomes compared with THA.

Interaction analyses indicated that the association between surgical method and outcomes differed by ASA class. For functional outcomes, interaction effects were most pronounced for hemiarthroplasty (HA) compared with THA, including maintenance of independent living (OR 2.8, CI 1.5–5.3) and maintenance of outdoor walking ability (OR 7.8, CI 4.0–15.2). For maintenance of outdoor walking ability, interaction was also observed for internal fixation (IF) (OR 2.8, CI 1.6–4.8), whereas no corresponding interaction was observed for IF in maintenance of independent living (OR 1.2, CI 0.65–2.2). For 1-year mortality, interaction was similarly observed for HA (OR 5.1, CI 2.0–12.6), but not for IF (OR 1.0, CI 0.38–2.7), indicating that the association between surgical method and outcome differed by ASA class, particularly for HA. Among patients with ASA I–II, THA was consistently associated with more favorable functional outcomes. In contrast, among patients with ASA III–IV, differences between surgical methods were attenuated, although outcomes generally remained more favorable after THA (Table 3).

**Table 3:** Functional outcome at 4 months and mortality at 4-month and 1-year follow-up for ASA III–IV patients treated with THA, IF, or HA. Values are count and (%) or unadjusted and adjusted odds ratio (OR) with 95% confidence interval (CI) using binary logistic regression analysis

	Total n	THA	IF	HA	IF vs THA OR (CI)	HA vs THA OR (CI)
Functional outcome at 4 months	676	314 (46)	144 (22)	218 (32)		
Maintained independent living	595 (88)	294 (94)	121 (84)	180 (83)	0.43 (0.22–0.86) **^a^**	0.43 (0.23–0.86) **^a^**
					0.42 (0.23–0.66) **^b^**	0.43 (0.23–0.79) **^b^**
Maintained outdoor walking ability	464 (69)	209 (69)	92 (68)	163 (82)	0.95 (0.62–1.4) **^a^**	2.0 (1.3–3.2) **^a^**
					0.96 (0.63–1.5) **^b^**	2.1 (1.3–3.2) **^b^**
Wheelchair dependence	58 (8.6)	24 (8.0)	15 (10)	19 (9.0)	1.4 (0.72– 2.8) **^a^**	1.1 (0.61–2.8) **^a^**
					1.4 (0.73–2.8) **^b^**	1.2 (0.62–2.2) **^b^**
Mortality	1,372	638 (47)	305 (22)	429 (31)		
At 4 months	89 (6.5)	24 (3.8)	27 (8.9)	38 (8.9)	2.5 (1.4–4.4) **^a^**	2.5 (1.4–4.4) **^a^**
					2.0 (1.1–3.7) **^c,d^**	2.5 (1.3–4.7) **^c,d^**
At 1 year	144 (11)	46 (7.2)	43 (14)	55 (13)	1.9 (1.3–2.9) **^a^**	2.1 (1.4–3.3) **^a^**
					2.9 (1.3–3.5) **^c,d^**	2.6 (1.5–4.3) **^c,d^**

For abbreviations, see Table 1.

a-c See Table 2.

d Missing = 383 (29%).

Among patients living independently and able to walk outdoors before fracture, those treated with THA more frequently maintained independent living and outdoor walking ability at 4 months compared with IF and HA. Wheelchair dependence was least common after THA (see Table 2). In adjusted logistic regression analyses (adjusted for age, sex, and ASA), both IF and HA were associated with lower odds of favorable functional outcomes than THA (see Table 2). Among patients with ASA I–II, THA was consistently associated with higher odds of maintaining independent living and outdoor walking ability, and with lower odds of wheelchair dependence, compared with both IF and HA (Table 4). In contrast, among patients with ASA III–IV, differences between surgical methods were attenuated, and THA did not confer better outdoor walking ability than IF or HA (see Table 3).

**Table 4:** Functional outcome at 4 months and mortality at 4-month and 1-year follow-up for for ASA I–II patients treated with THA, IF, or HA. Values are count and (%) or unadjusted and adjusted odds ratio (OR) with 95% confidence interval (CI) using binary logistic regression analysis

	Total n	THA	IF	HA	IF vs THA OR (CI)	HA vs THA OR (CI)
Functional outcome at 4 months	1,156	753 (65)	297 (25)	106 (10)		
Maintained independent living	1,098 (95)	736 (98)	281 (95)	81 (79)	0.43 (0.21–0.88) **^a^**	0.08 (0.04–0.16) **^a^**
					0.41 (0.20–0.86) **^b^**	0.09 (0.04–0.17) **^b^**
Maintained outdoor walking ability	950 (82)	670 (90)	216 (75)	64 (70)	0.35 (0.24–0.52) **^a^**	0.26 (0.16–0.43) **^a^**
					0.36 (0.25–0.51) **^b^**	0.26 (0.16–0.43) **^b^**
Wheelchair dependence	55 (4.8)	13 (2.0)	26 (9.0)	16 (16)	5.5 (2.8–11) **^a^**	10 (4.8–22) **^a^**
					5.5 (2.8–11) **^b^**	10 (4.9–23) **^b^**
Mortality	1,916	1,201 (65)	526 (27)	189 (10)		
At 4 months	58 (3.0)	16 (1.0)	27 (5.0)	15 (8.0)	4.0 (2.1–7.5) **^a^**	6.4 (3.1–13) **^a^**
					3.6 (1.8–7.5) **^c,d^**	3.9 (1.7–9.5) **^c,d^**
At 1 year	127 (6.6)	46 (4.0)	48 (9.0)	33 (18)	2.5 (1.7–3.8) **^a^**	5.3 (3.3–8.6) **^a^**
					2.5 (1.5–4.3) **^c,d^**	2.1 (1.3–3.5) **^c,d^**

For abbreviations, see Table 1.

a–c See Table 2.

d Missing 541 (29%).

## Discussion

We aimed to evaluate mortality and short-term functional outcomes in patients aged 60–69 years who underwent internal fixation (IF), hemiarthroplasty (HA), or total hip arthroplasty (THA) for displaced femoral neck fractures. The majority (56%) were treated with THA. Our findings indicate a clear selection pattern: patients with cognitive impairment, reduced pre-fracture mobility, or greater comorbidity were more likely to receive HA, and these groups also demonstrated higher 1-year mortality. THA was associated with superior short-term functional outcomes, whereas this benefit was not observed in the more comorbid ASA III–IV group. Overall, our results emphasize the importance of individualizing surgical choice in hip fracture patients, balancing expected long-term functional gains from THA against perioperative risk and rehabilitation capacity, particularly in patients with high ASA scores. Together, these findings suggest that ASA class is associated with the functional benefit of THA—patients with higher physiological reserve can benfit most from the procedure’s mechanical advantages, whereas in frailer patients a less demanding operation such as HA may better support short-term mobility.

Our findings are partially consistent with previous observational studies. A Swedish cohort study reported no statistical differences in patient satisfaction or mortality between IF and THA [18]. Likewise, analyses from the Norwegian Hip Fracture Register found only small differences in PROMs and no difference in 1-year mortality between THA and IF in patients aged 55–70 years [19]. However, our study demonstrated superior short-term functional outcomes after THA, particularly among patients with ASA I–II, and lower adjusted mortality compared with IF. These differences may reflect variation in study populations, measured functional outcomes, adjustment for confounding, and analytical methods.

Prior studies variably use health-related quality of life (EQ-5D) or condition-specific functional measures, while others focus on clinical outcomes like ours [20,21]. Regardless of the methods used, baseline differences require careful consideration when interpreting the outcome.

Confounding by indication was evident in our study, and differences in baseline characteristics between the treatment groups were also observed in another study from the Swedish Hip Registry of patients aged 60–69 years [18]. In that study the baseline age, sex, mobility, and comorbidity resemble ours, supporting our baseline results with poorer health and function in HA recipients [18]. These findings align with Swedish practice recommendations favoring HA in older, more comorbid patients, THA in healthier older patients, and IF in younger patients [22]. Similar selection has been reported in another study in patients aged 55–70 years [19].

Mortality analyses yielded somewhat different patterns depending on the analytical approach. While adjusted logistic regression identified differences in mortality between treatment groups at fixed time points, Kaplan–Meier analyses showed comparable survival among patients with ASA I–II but lower survival after HA and intermediate survival after IF compared with THA among patients with ASA III–IV. These findings reflect the different aspects of mortality assessed by the 2 statistical methods rather than contradictory results. Among patients with ASA I–II, absolute mortality was low across all surgical methods, and the survival curves largely overlapped throughout follow-up, with overlapping confidence intervals. This suggests that any differences in mortality between surgical methods were small and of uncertain clinical relevance in this subgroup.

In contrast, among patients with ASA III–IV, survival probabilities differed more clearly between surgical methods throughout follow-up, with consistently lower survival after HA and intermediate survival after IF compared with THA. These findings were consistent with the adjusted regression analyses and suggest a stronger association between treatment and mortality in this higher-risk population.

Residual confounding by indication is also likely to contribute. Although ASA stratification improves comparability, patients selected for THA within each ASA category may still represent a healthier subset with greater physiological reserve, particularly among those classified as ASA III–IV. High ASA and dementia are established mortality predictors after hip fracture and should be accounted for [23]. The observation of lower mortality among ASA class III–IV patients who underwent THA is intriguing and, to our knowledge, not well described in prior literature. Most registry and cohort studies report that higher ASA (or frailty indices) predict increased mortality after hip fracture, and while some adjusted analyses find lower mortality with THA vs hemiarthroplasty overall, these studies do not present ASA-stratified results [24,25]. Therefore, our finding is most likely explained by selection bias (healthier patients within ASA III–IV being selected for THA), center, or surgeon effects, or other confounding perioperative factors. Prior studies rarely provide ASA-stratified estimates [24,25], and 1 review suggested that mortality is more strongly associated with age and comorbidity than with surgical method [26].

Among patients with ASA I–II, THA was associated with superior functional recovery at 4 months, including higher rates of maintenance of independent living and outdoor walking ability and less wheelchair dependence. These findings are consistent with the greater biomechanical stability and pain relief associated with THA compared with IF and HA, benefits that may be most effectively exploited by patients with sufficient physiological reserve and rehabilitation capacity.

In contrast to our findings, Lagergren et al. [18] reported no difference in functional outcomes between IF and THA at 1 year. However, their study included a substantially smaller cohort, excluding patients treated with HA, and lacked adjustment for important baseline characteristics such as ASA class.

Conversely, among patients with ASA III–IV, functional differences between surgical methods were smaller and were largely attenuated after adjustment. This suggests that, in patients with greater comorbidity, postoperative recovery is influenced predominantly by underlying health status rather than by the choice of surgical technique.

### Strengths

We had a large, nationally representative cohort with high registry coverage (80–86%) [8] and evaluated clinically relevant outcomes, including mortality, residence, and mobility. We adjusted for important potential confounders, including age, sex, ASA classification, and cognitive status.

### Limitations

Treatment allocation was not randomized but strongly influenced by baseline health, functional status, and comorbidity, introducing a risk of residual confounding. Although ASA classification was used to improve comparability, it may not fully capture differences in physiological reserve. Other limitations include the relatively short functional follow-up (4 months) inherent to SHR and the substantial non-response rate at follow-up. Non-responders were frailer and more dependent at baseline, suggesting that absolute levels of functional recovery may be overestimated. However, as the proportion and baseline characteristics of non-responders were similar across surgical methods, the risk of differential selection bias affecting comparisons between THA, IF, and HA is likely limited (see Figure 1).

Finally, group sizes differed between treatments, with fewer patients in the HA and IF groups than in the THA cohort. This may affect the precision of some subgroup estimates. While residual selection bias related to non-response and limited subgroup size may influence the precision of some estimates, the overall patterns were consistent across analyses and aligned with findings from previous studies.

### Conclusion

In patients with displaced femoral neck fractures, THA was associated with more favorable short-term functional outcomes than IF or HA, primarily among patients with lower comorbidity (ASA I–II). Mortality patterns varied across ASA category, with differences between surgical methods being smaller among patients with lower comorbidity and more pronounced among those with higher comorbidity.

Given the observational design and potential for selection bias, these findings should be interpreted with caution.

*In perspective,* these findings support individualized treatment decisions based on physiological reserve rather than age alone and caution against attributing mortality differences in observational data to surgical method per se, in accordance with a recent educational article [6].

### Supplementary data

Supplementary Tables S1–S3 are available as supplementary data on the article homepage, doi: 10.2340/17453674.2026.46368

## Supplementary Material



## Notes

MH, PC, AA, and OO conceived and designed the study. MH and OO were responsible for patient inclusion and data collection. AA performed the statistical analyses. MH, KM, AA, and PC drafted the manuscript and critically revised it for important intellectual content. All authors approved the final version of the manuscript and agree to be accountable for all aspects of the work.

The authors thank statistician Orestes Theodoridis for statistical support.

Handling co-editors: Taco Gosens and Robin Christensen

*Acta* thanks Pieter Joosse and Lene Bergendal Solberg for help with peer review of this manuscript.
