# Supplementary material for: Association of treatment method with mortality and functional outcome in patients with displaced femoral neck fracture: a cohort study of 60–69-year-old patients from the Swedish Hip Fracture Register
Source: Acta Orthop. 2026 Sep 3;97:615–22. doi: 10.2340/17453674.2026.46368 (PMC13543153; doi:10.2340/17453674.2026.46368)
Supplement: Supplementary file 1 [file ActaO-97-46368-s1.pdf]

Supplementary Tables

Table S1. Functional outcome at 4 months follow-up for patients operated with THA, IF or HA showing 4 months sample (n = 18,50) Data presented as n (%)

	**4 months' sample n = 1,850**			
	**THA**	**IF**	**HA**	**P value [d]**
	N = 1,073 (58)	N = 438 (24)	N = 314 (18)	
**Residency [a]**				<0.001
Maintained independent living				
Yes	1,036 (97)	407 (93)	265 (84)	
No	37 (3)	31 (7)	49 (16)	
**Walking ability**				<0.001
Maintained walking outdoor				
Yes	885 (84)	310 (72)	231 (78)	
No	172 (16)	118 (28)	64 (22)	
**Walking aid**				<0.001
Relay on wheelchair				
Yes	37 (3)	42 (9)	35 (11)	
No	1,034 (97)	403 (91)	290 (89)	

[a] Included n = 1,825, [b] included n = 1,780, [c] included n = 1,841, [d] Pearson chi-square test.
Abbreviations: IF = Internal fixation, HA = Hemiarthroplasty, THA = Total hip arthroplasty.

Table S2. Functional outcome at 4 months follow-up for patients operated with THA, IF or HA showing 4 months sample (n = 1,850). Data presented as n (%)

	ASA 1–2				ASA 3–4			
	**THA**	**IF**	**HA**	**P value [d]**	**THA**	**IF**	**HA**	**P value [d]**
**Residency [a]**								
Maintained independent living	N = 752	N = 294	N = 103	<0.001	N = 313	N = 138	N = 207	0.01
Yes	736 (98)	281 (96)	81 (79)		294 (94)	121 (88)	180 (87)	
No	16 (2)	13 (4)	22 (21)		19 (6)	17 (12)	27 (13)	
**Walking ability [b]**								
Maintained walking outdoor	N = 747	N = 288	N = 92	<0.001	N = 304	N = 136	N =1 99	0.002
Yes	670 (90)	216 (75)	64 (70)		209 (69)	92 (68)	163 (82)	
No	77 (10)	72 (25)	28 (30)		95 (31)	44 (32)	36 (18)	
**Walking aid [c]**								
Relay on wheelchair	N = 749	N = 296	N = 103	<0.001	N = 313	N = 143	N = 218	0.6
Yes	13 (2)	26 (9)	16 (16)		24 (8)	15 (10)	19 (9)	
No	736 (98)	270 (91)	87 (84)		289 (92)	128 (90)	199 (91)	

[a] Included n = 1,807, [b] included n = 1,766, [c] include n = 1,822, [d] Pearson chi-square test;
Abbreviations: IF = Internal fixation, HA = Hemiarthroplasty, THA = Total hip arthroplasty.

Table S3. Unadjusted OR for functional outcome at 4 months comparing patients 2 with different surgical methods using binary logistic regression analyses. Divided into 2 groups

	Unadjusted OR (CI)	
**Maintained independent living** [a]	ASA 1–2	ASA 3–4
THA (Ref)		
IF	0.47 (0.22–0.99)	0.46 (0.23–0.91)
HA	0.08 (0.04–0.16)	0.43 (0.23–0.8)
**Maintained walking outdoor** [b]		
THA (Ref)		
IF	0.35 (0.24–0.49)	0.95 (0.61–1.47)
HA	0.26 (0.16–0.43)	2.06 (1.33–3.18)
**Relay on wheelchair** [c]		
THA (Ref)		
IF	5.5 (2.76–10.4)	1.41 (0.72–2.78)
HA	10.4 (4.85–22.4)	1.15 (0.61–2.16)

[a] Included n = 1,807, [b] included n = 1,766, [c] included n = 1,822

Abbreviations: IF = Internal fixation, HA = Hemiarthroplasty, THA = Total hip arthroplasty.

ASA = American Society of Anesthesiologists Classification. OR = odds ratio, CI = 95% confidence interval.
